# *Clostridioides difficile* infection leading to fulminant colitis with toxic megacolon

**DOI:** 10.4322/acr.2023.457

**Published:** 2023-11-16

**Authors:** Fareed Rajack, Shawn Medford, Tammey Naab

**Affiliations:** 1 Howard University Hospital, Department of Pathology and Laboratory Medicine, Washington, D.C., United States of America; 2 Howard University College of Medicine, Washington, D.C., United States of America

**Keywords:** Colectomy, Colitis, Colorectal Surgery, Diarrhea, Pseudomembranous

## Abstract

*Clostridioidesdifficile* infection (CDI) is the culprit of millions of nosocomial infections in the United States. Programs that successfully decrease its incidence, therefore, render cost savings for the healthcare system. Toxic megacolon and perforation are two of the most significant complications with increased mortality rates. We report a 23-year-old nursing home resident hospitalized for fever, cough, and green sputum. After 3 days of antibiotic therapy, he developed abdominal distension, diarrhea, and vomiting and underwent a total colectomy. The colon was dilated to a maximum of 11 cm with markedly edematous mucosa and yellow pseudomembranes. Qualitative PCR of the stool detected *Clostridioides difficile* toxin B gene. While there is no consensus for the required interval between antibiotic treatment and CDI, this presentation 3 days after starting the antibiotic therapy is earlier than most proposed ranges.

## INTRODUCTION

*Clostridioidesdifficile* (CD) colonizes 5% of adults and 15-70% of infants.^1^ In the United States (US), *Clostridioides difficile* infection (CDI) affects up to 3 million patients annually. Up to 8% of hospitalized patients can develop CDI.^2^ Costs attributed to CDI amount to $4.8 billion annually since CDI is the most common cause of nosocomial infection in US hospitals, accounting for 15% of infections.^3-5^

The transmission route for CDI is fecal-oral and is often attributed to inadequate hand hygiene, leading to healthcare personnel transferring spores to patients. The three toxins associated with CDI are toxin A (enterotoxin A), toxin B (cytotoxin B), and *C. difficile* transferase (CDT or binary toxin). Studies have shown CDI development caused by each of the three toxins independently. These toxins and enzymes, including collagenase, hyaluronidase, and chondroitin-sulfatase, work together to cause cytoskeletal damage and, eventually, the compromised structure and functionality of the gut barrier.^1^

The manifestations of CDI can vary widely. Some patients are asymptomatic, while others may experience mild diarrhea and recover after 5-10 days of antibiotic therapy.^1^ 25% of patients with CDI may develop CD-associated diarrhea.^2^ The most severe cases of CDI manifest with significant complications, including systemic inflammatory response syndrome (SIRS), septicemia, intestinal paralysis, toxic megacolon, and colonic perforation.^1^ 0.4-3% may progress to fulminant CD colitis with toxic megacolon, which has a high mortality rate (38-80%) and requires prompt surgical intervention.^6^

The most common risk factors for CDI are increased age, medical comorbidities, hospitalization, and antibiotic therapy. Increased hospital length of stay is also a significant risk factor for CDI. Due to these factors, hospitalized patients and nursing home residents have the highest risk of developing CDI.^1^ A systemic review and meta-analysis of 13 studies found a CDI incidence of 8.3 cases per 10,000 patient-days.^4^ It also showed an upward trend in the ranges of incidence over the past 2 decades; per 10,000 patient-days, the CDI case incidence for studies from 2000-2008, 2008- 2009, and after 2010 were 2.8 to 12.2, 6.3 to 9.6, and 6.8 to 15.8, respectively.^4^

It has been shown that community-acquired CDI accounts for 30% of all cases of CDI.^1^ Furthermore, 2018 data from the CDC reports an incidence of 65.93 per 100,000 for community-associated CDI compared to 64.19 per 100,000 for healthcare-associated CDI.^7^ A retrospective cohort study utilizing the Healthcare Cost and Utilization Project (HCUP) database of US non-federal acute care hospitals showed that while hospital-acquired CDI decreased by 15.9% from 2006 to 2015, community-acquired CDI increased by 49.3% over the same period.^3^

This rise in community-acquired CDI cases has been attributed to low-risk patients who are younger and healthier without common risk factors such as recent hospitalizations and antibiotic use.^3^ It has been suggested that “traditional risk factors” may not be as significant for community-acquired CDI. Some explanations include diverse sources of infection (such as food, water, and animals) and genome fluidity leading to the genetic diversity of *C. difficile*.^8^

Antibiotics are regarded as “the most modifiable risk factor” for CDI. Variables associated with this risk are the duration of antibiotic treatment, the class of antimicrobial agent, and the number of antibiotic agents administered. An analysis of nursing home residents showed that compared with 7-day courses, 10-day courses had a 12% increased risk of CDI, and 14-day courses had a 27% increased risk of CDI. Given that cephalosporins, fluoroquinolones, and clindamycin are often associated with CDI infection, it computed the absolute risk ratio (ARR) of moxifloxacin to amoxicillin as 2.21, ciprofloxacin to nitrofurantoin as 1.89, and clindamycin to cloxacillin as 2.12.^9^ A study of hospitalized adults receiving antibiotics for at least 2 days showed that compared to those receiving one antibiotic, the adjusted hazard ratio (HR) was 2.5 for those administered 2 antibiotics, 3.3 for those administered 3 or 4 antibiotics, and 9.6 for those administered 5 or more antibiotics.^10^

## CASE REPORT

A 23-year-old male nursing home resident with a history of remote anoxic brain injury secondary to ventricular fibrillation arrest was hospitalized for evaluation of fever associated with cough and greenish sputum. He had developed abdominal distention with diarrhea and vomiting immediately before admission. He had a three-day history of antibiotic (linezolid, piperacillin, and tazobactam) use.

**Figure 1:**
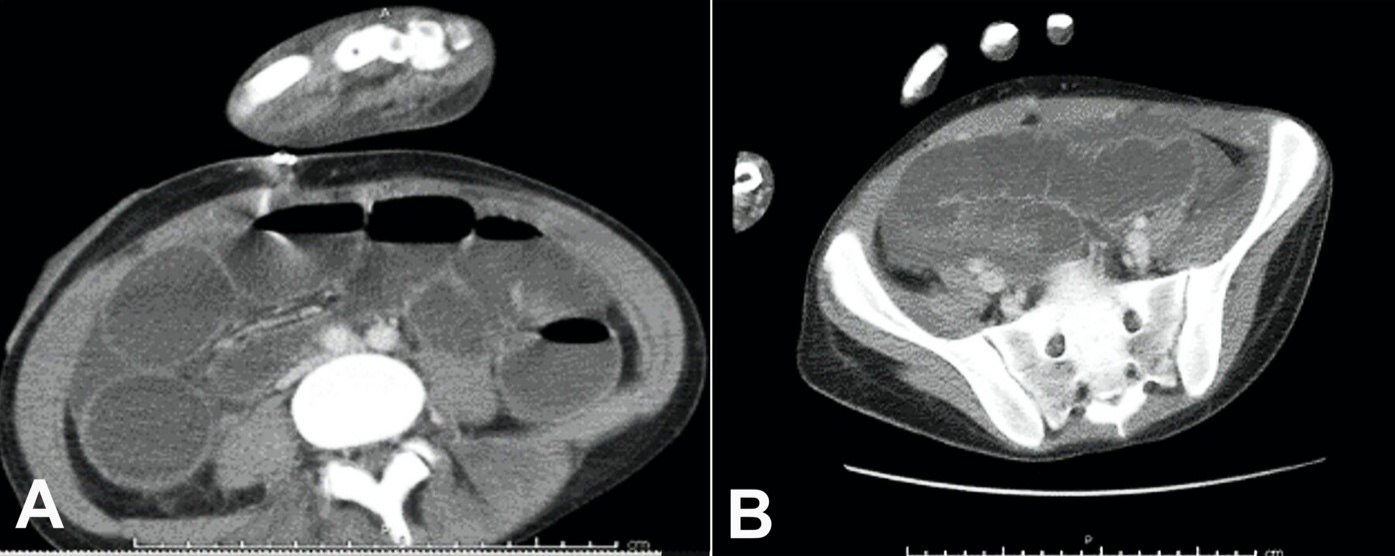
**A –** Non-contrasted abdominal computerized tomography showing dilated loops of bowel measuring 11 cm at maximum in the cecum and ascending colon; **B -** Non-contrasted pelvic computerized tomography showing abnormal bowel wall thickening with a maximum bowel thickness of 1.2 cm in the ascending colon.

On admission, his temperature, respiratory rate, and pulse were elevated to 39.6^o^C, 34/min, and 126/min, respectively. His white blood cell count was 19.65 cells/μL (neutrophil predominance of 90%). The abdomen was mildly distended but nontender. Stool analysis revealed no ova, cyst, or parasite. Stool lactoferrin test for neutrophils was positive. CT scan of the abdomen showed marked thickening in the wall of the ascending colon (Figure 1).

**Figure 2:**
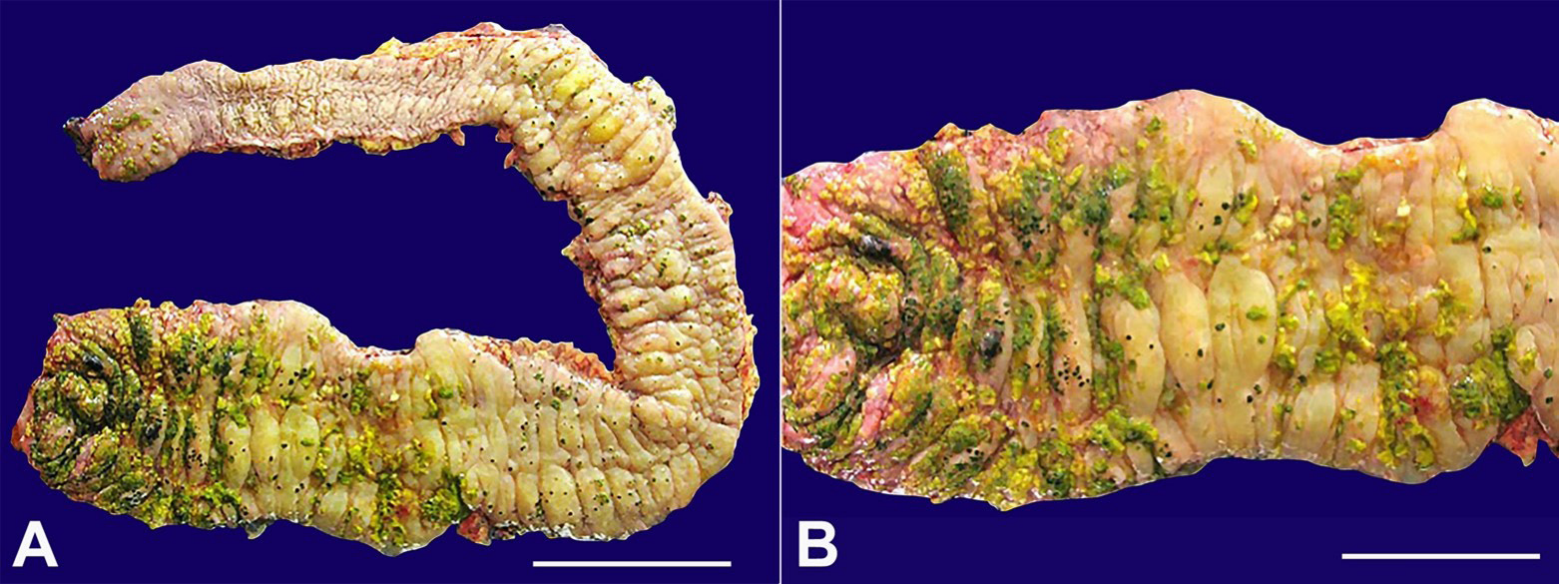
**A** and **B -** Gross view of the resected colon showing yellow-green plaques (pseudomembranes) adherent to the mucosal surface predominantly in the cecum and the right colon.

He underwent a total colectomy. The colon was grossly dilated, with a maximum of 11 cm dilation in the cecum and ascending colon. The mucosa was edematous, with a maximum bowel thickness of 1.2 cm in the ascending colon. Yellow pseudomembranes were observed throughout the colon but were primarily clustered in the cecum and ascending colon (Figure 2).

**Figure 3:**
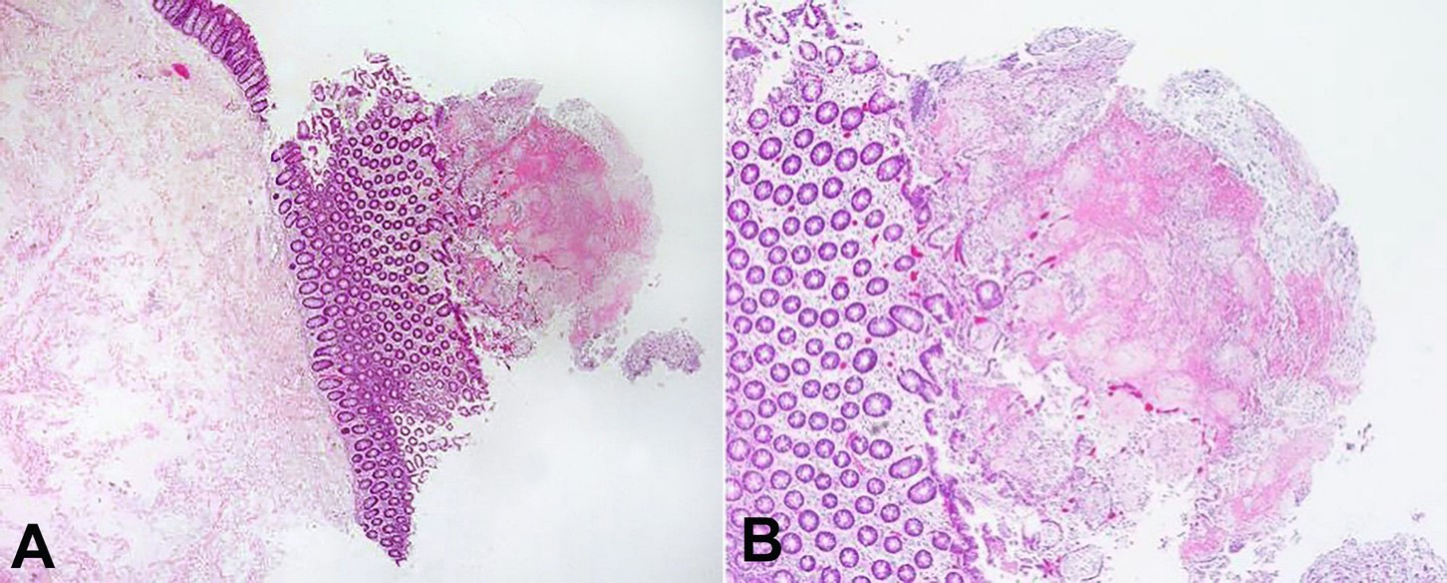
**A** and **B -** Histopathologic features of the resected colon: type I lesion, characterized by superficial mucosal necrosis with an erupting spray of fibrinopurulent exudate [H&E, 100x (A), 200x (B)].

The histologic changes were consistent with type I lesion, characterized by superficial mucosal necrosis with an erupting spray of fibrinopurulent exudate (Figure 3). *Clostridioides difficile* toxin B gene was detected in the stool sample by qualitative PCR.

The patient had no significant postoperative complications and was discharged to the nursing facility.

## DISCUSSION

CDI is caused by toxins A, B, and *C. difficile* transferase (CDT or binary toxin) acquired by a fecal-oral route but also from the environment as a form of nosocomial infection. The number and severity of CDIs can likely be attributed to the emergence of more pathogenic strains, especially the B1/NAP1/O27 strain, producing large amounts of CD toxins A and B.^11,12^

Toxic megacolon (TM) is a complication in 4.3% of CDI.^13^ It has been characterized by Jalan et al.^14^ as having 1) dilated colon over 6 cm, 2) either fever over 38.6 °C, tachycardia over 120 beats per minute, leukocytosis over 10,500 cells/µL, or anemia, and 3) either electrolyte disorders, hypovolemia, hypotension, or altered mental status.^13,14^ Inflammatory bowel disease (51.6%), septicemia (10.2%), and intestinal infection (4.1%) account for the leading causes for hospital admission.^13,15^

Toxic megacolon is postulated to develop from inflammation that penetrates the muscularis propria, leading to neural injury, altering gut motility, and resulting in dilation.^6^ Additionally, it is thought to be affected by altered colon response to chemical mediators, leading to impaired contraction of smooth muscles and lower basal luminal pressure. However, there is no consensus on the precise mechanism.^13,14,16-19^

The most common clinical manifestations are bloody diarrhea, hypotension, tachycardia, fever, and abdominal tenderness.^13^ Treatment is focused on addressing the underlying cause, centered on reducing inflammation to address colon motility and prevent perforation. Conservative and non-pharmacologic measures include fluid replacement, management of electrolyte derangement, bowel rest, nasogastric decompression, and encouragement of ambulation. Close observation and medical management are crucial, including monitoring labs (complete blood count and electrolytes), abdominal x-ray, maneuvers to redistribute colonic gas, and enteral feeding upon improvement. Glucocorticoids are the first-line therapy for TM in patients with inflammatory bowel disease (IBD) without evidence of impending colonic perforation. Otherwise, infliximab may be given, after three days, if there is insufficient response to glucocorticoids.^13^ The overall mortality rate for hospitalized patients with TM has been reported to be 7.9%.^13,15,20^ For those with colonic perforation, the mortality rate is 3-5 times higher.^13,21,22^

Fulminant *C. difficile* colitis has been defined as *C. difficile* colitis with systemic effects and shock leading to colectomy, intensive care unit admission, or death.^23^ It has previously been called severe, complicated CDI and has hypotension, shock, ileus, or megacolon as its characteristic features.^24^ The most significant complications of CDI are toxic megacolon and perforation.^25^ A retrospective review of 4,796 patients showed that fulminant CDI accounted for 4.1% of patients with *C difficile* colitis and had an in-hospital mortality rate of 34.7%.^23^

Age >75 years, ischemic or infectious colitis, malignancy, organ transplantation, immunosuppression, and diabetes mellitus are risk factors for fulminant colitis with toxic megacolon (FCTMC).^3,26^ In a young patient, inflammatory bowel disease is the most common cause of FCTMC. It is most often heralded by abdominal pain, abdominal distension, voluminous diarrhea with a distinctive odor, and characteristic lab findings, including granulocytic leukocytosis >15,000 cells/μL, serum creatinine >1.5X baseline, and serum albumin <3 g/dL.^26^

Perforation is the leading cause of mortality. 44% of cases of *C. difficile* colitis with perforation required surgery compared to 2% of cases without perforation.^13,27^ Signs of perforation include abdominal distension, rebound tenderness, and hemodynamic instability.^13^ Patients submitted to surgical intervention before perforation have an 8% mortality risk compared to 40% for those with surgery following colon perforation.^13,28^

Our patient, without evidence of IBD, was at risk due to antibiotic therapy and residence in a nursing home. The Infectious Diseases Society of America (IDSA) considers the highest-risk antibiotics for CDI to be third and fourth generation cephalosporins, fluoroquinolones, carbapenems, and clindamycin.^29^

A review of 528 cases of CDI found that 20 of the patients (3.7%) had a colectomy for fulminant *C. difficile* colitis (FCDC). 100% of these colectomy patients were given antibiotics before developing symptoms, and 45% were given multiple antibiotics. 35% (7 patients) received three prophylactic doses of cefuroxime before surgery and then progressed to FCDC. The authors recommended a single dose rather than three doses for prophylactic antibiotics prior to surgery and to avoid unnecessary use of cephalosporins.^30^ Another study showed that occupying the bed of a prior patient who received antibiotics led to an increased risk of CDI (hazard ratio of 1.22).^31^

Our patient developed colitis only three days after being treated with antibiotics. Most studies show that the median incubation period is less than 7 days.^32^ The IDSA guidelines on CDI infection in adults point out three studies showing 2-3 days to be the duration between exposure and CDI.^24,33-36^ More recent studies suggest the incubation period could be longer.^29^ Symptoms most commonly present after less than 48 hours of infection, but can also take 2-3 months to appear.^37,38^ This variability justifies that some advocate for CDI to be a non-reimbursable hospital service.^37^

The IDSA’s recommendation for treating fulminant CDI is 500 mg of vancomycin 4 times daily orally or via nasogastric tube. For patients with ileus, an alternative is a retention enema of 500 mg of vancomycin in 100 mL of normal saline administered rectally every 6 hours. 500 mg of intravenous metronidazole every 8 hours should also be added for patients with ileus in addition to vancomycin.^36^

Colectomy has been the standard therapy for FCTMC, but an alternative is diverting ileostomy with colonic lavage using vancomycin and metronidazole.^11,39^ The IDSA’s recommendation for surgical management is subtotal colectomy with rectal preservation or diverting loop ileostomy with colonic lavage and anterograde vancomycin flushes.^36^

Fidaxomicin has received much praise for reducing CDI recurrence while not disrupting normal colonic flora. A meta-analysis of 2,303 patients with CDI showed that fidaxomicin when compared to vancomycin, was associated with a lower CDI recurrence rate (OR 0.47, 95% CI 0.37-0.60) but was not associated with a different cure rate (OR 1.22, 95% CI 0.93-1.60).^40^ One study showed a CDI recurrence rate of 15.4% after fidaxomicin versus 25.3% after vancomycin.^1,41^ Another multicenter study of 535 patients with acute CDI demonstrated that fidaxomicin may be a viable alternative to vancomycin due to its comparable efficacy and safety. It showed that clinical cure (diarrhea resolution and no additional treatment required) was achieved by 91.7% of patients receiving fidaxomicin (200 mg per oral every 12 hours) and 90.6% of patients receiving vancomycin (125 mg per oral every 6 hours).^42^ However, fidaxomicin has not demonstrated the ability to reduce the recurrence of CDI infection with the BI/NAP1/027 strain.^1,41,42^

Fecal microbiota transplantation (FMT) has also shown impressive results in preventing recurrent CDI. A 2014 study of 27 patients given fecal filtrate directly into the intestines and a 2016 study of 20 patients given freeze-dried oral capsules demonstrated 100% effectiveness.^1,43,44^ Another study of 57 patients with severe CDI and fulminant CDI (based on the American College of Gastroenterology guidelines) refractory to antimicrobial therapy showed an overall 91% cure rate at 1 month with 100% for severe CDI patients and 87% for fulminant patients. It also demonstrated a 94.7% survival rate at 1 month and a 78.6% survival rate at 3 months. These results and the absence of adverse effects or perforation suggested that using FMT instead of colectomy could be a consideration for select patients.^45^ However, the FMT procedure is yet to be standardized, and there is concern about infection transfer and the development of autoimmune disorders influenced by the gut microbiome.^1^

The chromosomal pathogenicity locus is composed of *tcdA* and *tcdB* (genes encoding toxins A and B), *tcdC* and *tcdD* (regulatory genes), and *tcdE* (porin gene).^46^ A third toxin is *C. difficile* transferase (CDT), also called binary toxin, which is encoded by *cdtA* (disruption of actin filament assembly) and *cdtB* (cell-surface binding and intracellular translocation.^46,47^

The prevalence of the hypervirulent strain, BI/NAP/027, has increased significantly since 2000. BI/NAP1/027 is caused by mutations in the toxin regulatory gene (*tcdC*), specifically an 18-base pair deletion and position 117 deletion. This leads to the strain producing higher amounts of toxin A and toxin B as well as being resistant to fluoroquinolones.^1,48,49^ A characterization of isolates from 124 patients in Quebec along with US, UK, and other Canadian isolates for increased genetic diversity found that toxin A production was 16 times higher and toxin B production was 23 times higher for the NAP1/027 strain.^39,50^

In a historical database of over 6,000 isolates from 1984-1990, 18 isolates (less than 0.3%) from 14 patients were BI/NAP1. By contrast, out of 187 isolates collected since 2001 from eight healthcare facilities with *C. difficile*-associated outbreaks between 2000-2003, 96 isolates (51%) were BI/NAP1. Given the strain’s resistance to fluoroquinolones, it is likely that US hospitals’ increased use of fluoroquinolones has provided an opportunity for this hypervirulent strain to become much more prevalent in the past two decades.^1,49^

Pseudomembranous colitis (PMC) is characterized by pseudomembranes visualized endoscopically as yellow-white plaques measuring up to two centimeters in diameter on the mucosa of the colon and composed of dead intestinal cells, fibrin, and neutrophils.^1,51^ This normally appears as a diffuse pattern of scattered pseudomembranes throughout the mucosa. Confluent pseudomembranes on the entire mucosa may be seen in the most severe cases, and 3-8% of cases progress to a fulminant infection.^52^ PMC is reported to be the most common endoscopic finding in patients with CDI and is estimated to occur in 10% of patients with antibiotic-associated diarrhea.^51,53^

Antibiotics often alter the natural colonic flora, leading to *C. difficile* colonization. This or another inciting event causes endothelial damage, leading to necrotic areas on the surface epithelium, followed by inflammation.^51^ The influx of neutrophils and macrophage/monocyte activation leading to pro-inflammatory cytokine (e.g., IL-1, IL-9, TNF, LTB4) release causes pseudomembrane formation and mucosal injury.^51,54,55^ Both toxins and enzymes (i.e., collagenase, chondroitin-sulfatase, hyaluronidase) damage the cytoskeleton and cause the gut barrier to lose function. The cytoplasmic Rho protein (family of GTPases) is responsible for actin polymerization, stabilizing the cytoskeleton. Toxins of *C. difficile* inactivate Rho, leading to destabilized cytoskeleton, inflammation, and pseudomembrane formation or microulcerations in severe cases.^1,56,57^

## CONCLUSION

Currently, CDI is the most common healthcare-associated infection. It is mandatory to isolate CDI patients and to thoroughly clean environmental surfaces with a chlorine-based disinfectant to prevent spread.^53^ Hand hygiene and wearing gloves are crucial because the hands of healthcare providers are the main transmission mechanism. If a provider does not wear gloves and works with a patient with CDI, there is a 14-59% chance of hand contamination.^34,36,58,59^ Compliance with soap and water handwashing protocols for 15 to 30 seconds has been shown to be 20-40% effective.^32,60^

C. difficult spores are resistant to alcohol-based hand sanitizers. Handwashing using soap and water for 15 to 30 seconds (which remove but do not kill spores) is more effective in reducing spore burden.^61^ The ”Cleanyourhands” campaign consisted of alcohol-based hand rub installed at the bedside, hand hygiene promotion, and hygiene audits among 187 acute trusts in England and Wales from 2004 to 2008. It led to triple the hospital procurement of liquid soap and alcohol hand rub from 21.8 to 59.8 mL per patient bed day and was associated with a CDI incidence rate ratio of 0.80 (95% CI 0.71 to 0.90).^61,62^

The rapid incubation period for CDI leads to a risk of rapidly developing symptoms and the progression to severe complications, especially for high-risk patients. Patients are at higher risk for CDI for over three months since the use of antibiotics disrupts normal gastrointestinal flora. They may be prone to develop CDI during subsequent hospitalizations within this period.^63^

We are witnessing a shift in the standard of care from metronidazole and vancomycin to fidaxomicin and fecal microbiota transplantation. A study based on 2018 IDSA treatment guidelines found that the most cost-effective treatments for CDI were fidaxomicin for the initial treatment of non-severe cases, vancomycin for severe cases, fidaxomicin for the first recurrence, and FMT for subsequent

 recurrences.^64^
